# The influence of a single water molecule on the reaction of BrO + HO_2_

**DOI:** 10.1038/s41598-023-28783-x

**Published:** 2023-08-10

**Authors:** Peng Zhang, Lu Ma, Meilian Zhao, Yuxi Sun, Wanping Chen, Yunju Zhang

**Affiliations:** 1 Key Laboratory of Photoinduced Functional Materials, Key Laboratory of Inorganic Materials Preparation and Synthesis, Mianyang Normal University, Mianyang, 621000 People’s Republic of China; 2 College of Medical Technology, Chengdu University of Traditional Chinese Medicine Liutai Avenue, Wenjiang District, Chengdu, People’s Republic of China

**Keywords:** Physical chemistry, Theoretical chemistry

## Abstract

The influence of a single water molecule on the BrO + HO_2_ hydrogen extraction reaction has been explored by taking advantage of CCSD(T)/aug-cc-pVTZ//B3LYP/6-311 +  + G(d,p) method. The reaction in the absence of water have two distinct kinds of H-extraction channels to generate HOBr + O_2_ (^1^Δ_g_) and HBr + O_3_, and the channel of generation of HOBr + O_2_ (^1^Δ_g_) dominated the BrO + HO_2_ reaction. The rate coefficient of the most feasible channel for the BrO + HO_2_ reaction in the absence of water is estimated to be 1.44 × 10^–11^ cm^3^ molecule^−1^ s^−1^ at 298.15 K, which is consistent with the experiment. The introduction of water made the reaction more complex, but the products are unchanged. Four distinct channels, beginning with HO_2_^…^H_2_O with BrO, H_2_O^…^HO_2_ with BrO, BrO^…^H_2_O with HO_2_, H_2_O^…^BrO with HO_2_ are researched. The most feasible channels, stemming from H_2_O^…^HO_2_ with BrO, and BrO^…^H_2_O with HO_2_, are much slower than the reaction of BrO + HO_2_ without water, respectively. Thus, the existence of water molecule takes a negative catalytic role for BrO + HO_2_ reaction.

## Introduction

Methyl bromide stems from nature and humanity. It is the main precursor of active bromine involved in stratospheric ozone chemistry^1,2^. Bromine containing molecules, especially bromine oxide species are known to play a significant role in stratospheric ozone destruction and polar ozone hole chemistry^3,4^, in spite of their concentration being much lower than that of chlorine containing molecules. Such destruction takes place passing through catalytic cycles, in which the active substances are regenerated. In order to comprehend and simulate atmospheric ozone concentration, it is necessary to obtain the parameters describing the kinetics and photochemistry of these cycles.

Because the reaction of BrO + HO_2_ is of great significance in evaluating the influence of bromine on the damage of O_3_, it has attracted great interest of many research groups^3–10^. Yung et al.^5^ researched that the reaction of BrO + HO_2_ could induce ozone destruction cycle through synergistic coupling, and result in the generation of HOBr. The photolysis of HOBr could produce OH, and then OH reacts with ozone to complete the cycle (1–4). Interestingly, this cycle does not require the participation of oxygen atoms. Thus, the cycle of HO_2_ + BrO is of special importance in the lower stratosphere^3,5^.

1 $${\text{BrO}} + {\text{HO}}_{2} \to {\text{HOBr}} + {\text{O}}_{2}$$

2 $${\text{HOBr}} + hv \to {\text{OH}} + {\text{Br}}$$

3 $${\text{OH}} + {\text{O}}_{3} \to {\text{HO}}_{2} + {\text{O}}_{2}$$

4 $${\text{Br}} + {\text{O}}_{3} \to {\text{BrO}} + {\text{O}}_{2}$$

5 $${\text{Net:}}\,\,2{\text{O}}_{3} \to 3{\text{O}}_{2}$$

The mechanism and kinetics for the reaction of HO_2_ + BrO have been researched within a certain temperature and pressure range experimentally and theoretically. In the point of view of experiment, Cox and Sheppard^6^ measured the kinetics of the BrO + HO_2_ by means of the modulated photolysis and molecular modulation/UV–visible absorption resulting in the value of the rate constants of ${0}{\text{.5}}_{{{ - 0}{\text{.3}}}}^{{{ + 0}{\text{.5}}}} \times {10}^{{ - 11}}$ cm^3^ molecule^−1^ s^−1^ at 303 K, 760 Torr. Bridier et al.^7^ and Poulet et al.^3^ respective obtained the higher values of (3.4 ± 1.0) × 10^–11^ and (3.3 ± 0.5) × 10^–11^ cm^3^ molecule^−1^ s^−1^ taking advantaging of the flash photolysis/UVvisible absorption method and the discharge flow reactor and mass spectrometry techniques. The HO_2_ + BrO reaction was studied again by the Larichev et al.^4^ at 233–344 K, and they obtained the Arrhenius expression of $k = \left( {4.8 \pm 0.3} \right) \times 10^{ - 12} \exp \left[ {\left( {580 \pm 100} \right)/T} \right]$ cm^3^ molecule^−1^ s^−1^. In 1996, Elrod et al.^8^ and in 1997, Li et al.^11^ also implemented in a discharge flow reactor with a mass spectrometer and demonstrated the negative dependence of the rate constant on temperature. The reported values of the reaction rate constant at 298 K were significantly lower in these studies than in earlier studies. Finally, results from the three most recent research of Cronkhite et al.^12^, Bloss et al.^9^ and Ward et al.^13^ where the HO_2_ + BrO reaction was investigated by means of the laser flash photolysis/UV absorption/IR tunable diode laser absorption, resulting in the rate constants at 296 K ($\left( {{2}{\text{.0}} \pm 0.{6}} \right) \times 10^{{ - 1{1}}}$ cm^3^ molecule^−1^ s^−1^), flash photolysis/time resolved UV absorption spectroscopy with the obtained rate constants at 298 K 760 Torr ($\left( {{2}{\text{.35}} \pm 0.{82}} \right) \times 10^{{ - 1{1}}}$ cm^3^ molecule^−1^ s^−1^), and by means of flash photolysis/Vis-UV absorption at 246–314 K with the arrhenius formula of $k = \left( {{9}{\text{.28}} \pm {5}{\text{.61}}} \right) \times 10^{ - 12} \exp \left[ {\left( {{2}{\text{.63}} \pm 1.{31}} \right)/\text{RT}} \right]$ cm^3^ molecule^−1^ s^−1^. In the computational work, Guha and Francisco^14^ researched the geometries and relative energies of the HOOBrO and HOOOBr generated from the BrO + HO_2_ reaction, and showed that HOOBrO and HOOOBr dissociated to HOBr + O_2_ and HBr + O_3_ with the barrier of 2.8 and 26.40 kcal/mol, respectively, which is consistent with the computed results in this work. In 2019, Tsona, Tang and Du researched ‘‘Impact of water on the BrO + HO_2_ gas-phase reaction: mechanism, kinetics and products^15^. The obtained results revealed significant differences from those published earlier on this reaction by Chow et al.^16^ In 2021, Chow et al.^17^ performed further calculation for this present work, combined with higher level calculations published by Chow et al.^16^, demonstrate that the work of Tsona et al. is flawed because the integration grid size used in their lowest singlet and triplet calculations is too small, and a closed-shell wavefunction, rather than an open-shell wavefunction, has been used for the singlet surface. The major conclusion in the work of Tsona et al. that the lowest singlet and triplet channels are barrierless is shown to be incorrect. Moreover, the calculated rate constants by Tsona et al. showed a positive temperature dependence, which is inconsistent with the experimentally observed negative temperature dependence, whereas the singlet rate constants for the BrO + HO_2_ → HOBr + O_2_ reaction which produces singlet O_2_ computed by Chow et al.^16^ revealed a negative temperature dependence consistent with experiment.

As is well known, there are a great quantity of water and water clusters in the atmosphere. Water could act as acceptor and donor in a hydrogen bond, and could form a hydrogen bond with active radicals and polar molecules. Hence, it could easily generate stable cyclic compounds with other species^18^. In recent years, more and more attention has been paid to the influence of water on the gas-phase reaction.^19–26^ Numerous theoretical and experimental studies have found that water molecules could decrease the reaction energy barrier^26–35^. Moreover, some researches revealed that water dimer could also take an significant catalytic role in H-abstraction reaction at 298 K at the atmospheric concentration of 9.0 × 10^14^ molecular cm^3^^36–39^. Thus, to fully comprehend this atmospheric process, it is necessary to further study the influence of water on BrO + HO_2_ reaction. High temperature reduces the stability of weak bond complexes in the lower troposphere, so this must also be borne in mind. Quantum chemical calculation can provide theoretical guidance for the study of such species.

In this work, the detailed channels of BrO + HO_2_ reaction on the singlet potential energy surface (PES) without water and containing water are researched using theoretical methods to establish the reaction mechanism and the influence of water according to the detailed potential energy surface.

## Computational method

Gaussian 09 program package^40^ was used to obtain all the results of the quantum chemical computations. B3LYP^41,42^ method combined with the 6-311 +  + G(d,p) basis set were employed to optimize and characterize all the species on the PESs. Harmonic vibrational frequencies were also gained at the same level to testify that transition states only possesses one imaginary frequency and other speices possess no imaginary frequencies, and the thermodynamic dedication to the free energy and enthalpy and the value of the zero-point energy (ZPE) at the identical level. Intrinsic reaction coordinate (IRC) computations^43,44^ was used to guarantee the linkage of the transition state between reactants and expected products. CCSD(T)^45^/aug-cc-pVTZ method was used to gained more accurate energy on account of the geometric configuration at B3LYP method. The rate coefficients of the BrO + HO_2_ reaction were employed by the KisThelP program^46^, which is based on the Transition State Theory (TST) with Wigner tunneling correction. According to the study of Shiroudi^47^, the detailed calculation process of rate coefficient is in the supporting information. In the following discussion, the B3LYP/6-311 +  + G(d,p) optimized geometric parameters and CCSD(T)/aug-cc-pVTZ + ZPE energies are used unless otherwise stated.

## Results and discussion

### The H-abstraction of the BrO + HO_2_ reaction with water-free

**Figure 1:**
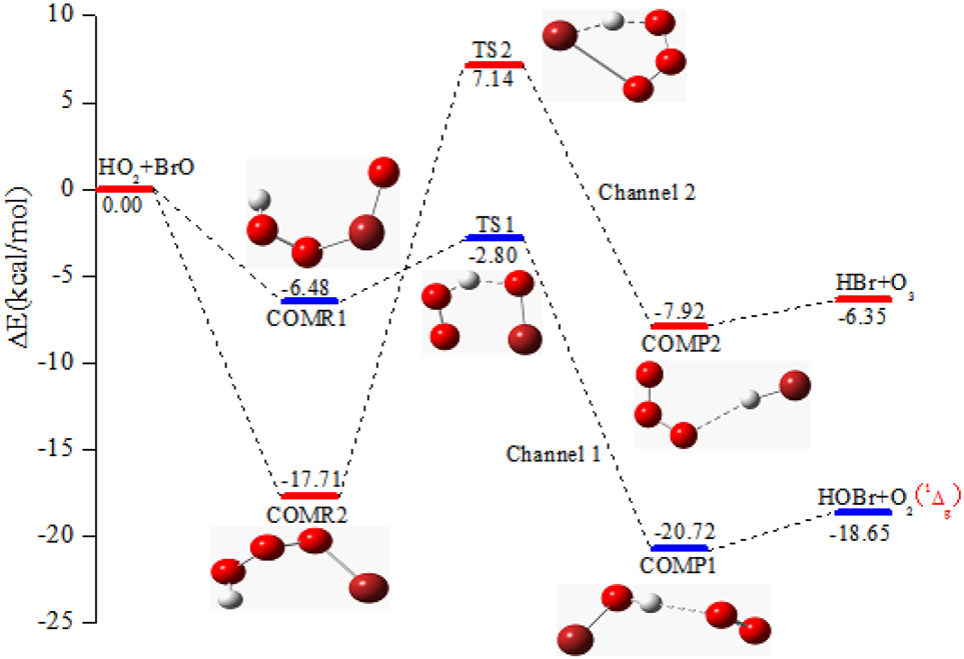
The potential energy surface for the HO_2_ and BrO reaction in the absence of water are calculated at the CCSD(T)/aug-cc-pVTZ//B3LYP/6-311 +  + G(d,p) level.

Similar to the previous investigations on the H-extraction reaction of BrO + HO_2_^14^, two distinct products channels of the generation of HBr + O_3_ and HOBr + O_2_ (^1^Δ_g_) were simulated located for the anhydrous BrO + HO_2_ reaction (see Fig. 1). Complex intermediate will be generated at the entrance and exit of these two pathways. As for the pathway of generation of HOBr and singlet O_2_, Channel 1 results in the generation of pre-reactive complex COMR1, and subsequently proceeds via TS1 with the forecasted energy of 2.80 kcal/mol (see Table 1) below BrO + HO_2_, to generate post-reactive complex COMP1. The barrier of COMR1 → TS1 → COMP1 is 3.68 kcal/mol, which is consistent with the results obtained by Guha and Francisco (2.80 kcal/mol)^14^. The energy of COMP1 with respect to the reactant are − 20.72 kcal/mol. In the channel of generation of HBr + O_3_ (Channel 2), pre-reactive complex COMR2 will be generated with no barrier from the combination of BrO with HO_2_. With respect to COMR2, the barrier of the generation of HBr + O_3_ is 24.85 kcal/mol, which is consistent with the results obtained by Guha and Francisco (26.40 kcal/mol). Stemming from COMR2, the reaction goes through TS2 to generate post-reactive complex COMP2 before generating the final products HBr and O_3_. COMP2 is steadied through the interaction of the hydrogen bond wth the binding energy of 7.92 kcal/mol below BrO + HO_2_. The Channel 1 is superior to the Channel 2 owning to the higher barrier height. In addition, the pathway on the triplet surface contribute less to the BrO + HO_2_ reaction due to the higher barrier height. Thus we have no further consideration in here.

**Table 1:** Relative energies (∆*E*), enthalpies (∆*H*) and Gibbs free energies (∆*G*) for the BrO + HO_2_ reaction are calculated at the CCSD(T)/aug-cc-pVTZ//B3LYP/6-311 +  + G(d,p) level.

Species	∆*E*_298K_	∆*H*_298K_	∆*G*_298K_
BrO + HO_2_	0.00	0.00	0.00
COMR1	− 6.48	− 7.11	3.23
TS1	− 2.80	− 3.93	7.58
COMP1	− 20.72	− 20.51	− 14.19
COMR2	− 17.71	− 18.58	− 7.65
TS2	7.14	6.02	17.18
COMP2	− 7.92	− 7.74	− 1.07
HBr + O_3_	− 6.35	− 6.36	− 4.98
HOBr + O_2_ (^1^Δ_g_)	− 18.65	− 18.62	− 17.36

All energies are computed with respect to the energy of BrO + HO_2_, (units: kcal/mol).

### The H-abstraction of the BrO + HO_2_ reaction with a water molecule

To assess the influence of a single water molecule on the H-extraction for the BrO + HO_2_ reaction in the atmosphere, distinct pathways have been investigated. Analogue to the aforementioned naked reaction, a pre-reactive complex will be generated at the beginning of each reaction channel with water. It should be mentioned that since it is impossible for the collision of three isolated molecules (including HO_2_, BrO and H_2_O) simultaneously, they will firstly generate a two-body complex, and then generate a three-body complex by the collision between the third specie and the two-body complex. Hence, in the existence of one water molecule, both BrO and HO_2_ could combine with the water molecule through hydrogen bond to firstly generate corresponding binary complexes before combining with the third species. Four hydrogen bonded complexes have been located, namely as BrO^…^H_2_O, H_2_O^…^BrO, H_2_O^…^HO_2_ and HO_2_^…^H_2_O. The energies of BrO^…^H_2_O, H_2_O^…^HO_2_ and HO_2_^…^H_2_O are − 2.84, − 6.79 and − 1.95 kcal/mol, which are consistent with the obtained results by Tsona et al. (− 2.51, − 6.51 and − 1.31 kcal/mol). The water moiety in BrO^…^H_2_O and HO_2_^…^H_2_O serve as a hydrogen bond donor, and water acts as both the H-bond acceptor and donor in H_2_O^…^HO_2_, as well as there exist one halogen bonded complex in H_2_O^…^BrO. The complex H_2_O^…^HO_2_ presents a five-membered-ring structure by generating two hydrogen bonds (2.641 Å and 1.773 Å, see Fig. S1), which are more stable than HO_2_^…^H_2_O, BrO^…^H_2_O and H_2_O^…^BrO by 4.84, 3.95 and 3.05 kcal/mol, respectively. Subsequently, these four binary complexes could further combine with the third species to generate three body complexes, and generate post-reactive complexes by surmounting corresponding transition state and then released to the final products. When a water molecule participates in the reaction, we found that, the reaction products are the same compared with anhydrous reaction, but the potential energy surface (PES) is complicated. In this paper, four pathways in the existence of water are employed to describe the influence of water molecule on the generation of HBr + O_3_ and HOBr + O_2_ (^1^Δ_g_) from the reaction of BrO + HO_2_ under atmospheric conditions.

#### The reactions of BrO + HO_2_^…^H_2_O and BrO + H_2_O^…^HO_2_

**Figure 2:**
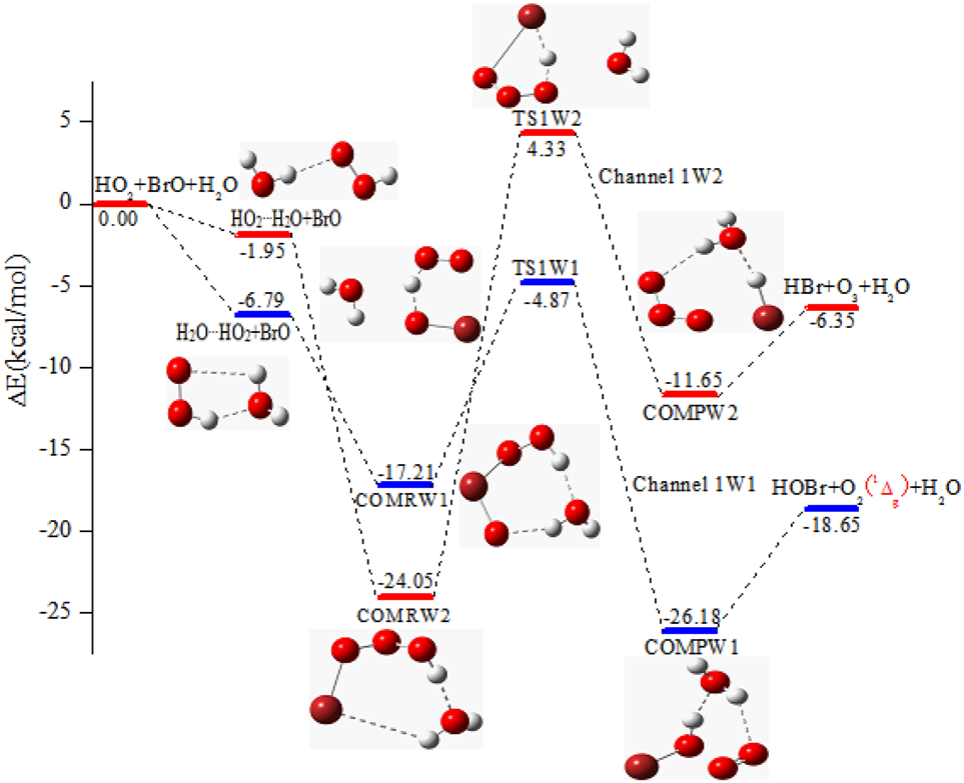
The potential energy surface for the HO_2_ and BrO reaction in the existence of water taking place via H_2_O^…^HO_2_ + BrO and HO_2_^…^H_2_O + BrO pathways are calculated at the CCSD(T)/aug-cc-pVTZ//B3LYP/6-311 +  + G(d,p) level.

In the existence of water, the channels on the PES for the generation of HOBr + O_2_ (^1^Δ_g_) and HBr + O_3_ taking place by through the reactions of H_2_O^…^HO_2_ + BrO (Channel 1W1) and HO_2_^…^H_2_O + BrO (Channel 1W2) are displayed in Fig. 2. The H_2_O^…^HO_2_ + BrO reaction starts from the generation of the pre-reactive complex COMRW1, and the stable energy with respect to the separate molecules is − 17.21 kcal/mol (see Table 2). Considering the geometry, complex COMRW1 is a seven-membered ring consisted of two parts, which are bound together through two hydrogen bonds (1.731 Å and 1.916 Å). Beginning with the complex COMRW1, the reaction proceeds through the transition state TS1W1 involving the O atom of the BrO part extracting the H atom of HO_2_ to generate the post-reactive complex COMPW1, and then COMPW1 quickly decomposes into HOBr + O_2_ (^1^Δ_g_) + H_2_O. the energy of COMRW1 and TS1W1 in the existence of water decreased by 10.73 and 2.07 kcal/mol, respectively. The barrier of COMRW1 → TS1W1 → COMPW1 is 12.34 kcal/mol with respect to COMRW1. From the perspective of the generated product, water hardly takes part in Channel 1W1, because its presence increases the potential barriers of the Channel 1 by 8.66 kcal/mol. This shows that water molecule creates adverse effect on the generation of HOBr + O_2_ (^1^Δ_g_) for the BrO + HO_2_ reaction.

**Table 2:** Relative energies (∆*E*), enthalpies (∆*H*), and Gibbs free energies (∆*G*) for the BrO + HO_2_ + H_2_O reaction taking place via HO_2_^…^H_2_O + BrO, H_2_O^…^HO_2_ + BrO, BrO^…^H_2_O + HO_2_ and H_2_O^…^BrO + HO_2_ are calculated at the CCSD(T)/aug-cc-pVTZ//B3LYP/6-311 +  + G(d,p) level.

Species	∆*E*_298K_	∆*H*_298K_	∆*G*_298K_
HO_2_ + BrO + H_2_O	0.00	0.00	0.00
HO_2_^…^H_2_O + BrO	− 1.95	− 1.98	3.46
H_2_O^…^HO_2_ + BrO	− 6.79	− 7.46	0.40
BrO^…^H_2_O + HO_2_	− 2.84	− 2.94	2.42
H_2_O…BrO + HO_2_	− 3.74	− 3.66	2.48
COMRW1	− 17.21	− 18.70	1.60
TS1W1	− 4.87	− 5.86	12.54
COMPW1	− 26.18	− 26.96	− 8.76
COMRW2	− 24.05	− 25.16	− 6.56
TS1W2	4.33	3.61	20.80
COMPW2	− 11.65	− 12.46	5.22
COMRW3	− 17.04	− 18.46	1.64
TS1W3	− 4.86	− 5.86	12.55
COMPW3	− 25.97	− 26.72	− 8.56
COMRW4	− 24.34	− 25.39	− 7.09
TS1W4	5.62	4.83	22.68
COMPW4	− 25.37	− 25.93	− 8.67
HBr + O_3_ + H_2_O	− 6.35	− 6.36	− 4.98
HOBr + O_2_ (^1^Δ_g_) + H_2_O	− 18.65	− 18.62	− 17.36

All energies are computed with respect to the energy of BrO + HO_2_ + H_2_O (units: kcal/mol).

As for the route initiating the HO_2_^…^H_2_O + BrO, the reaction primitively generates hydrogen bond complex COMRW2, whose structure is analogue to the above-mentioned COMRW1. According to the relative energy, the generation of three-body complexes COMRW2 through between BrO and HO_2_^…^H_2_O is superior to the generation of COMRW1 through combination between BrO and H_2_O^…^HO_2_. Similar to TS1W1, water molecule acts as the role of bystander for TS1W2. The barrier of generating of HBr + O_3_ through TS1W2 in the existence of water is 3.53 kcal/mol higher than that without water. Similar to the way of generating HOBr + O_2_ (^1^Δ_g_), the existence of water molecules raises the barrier height, resulting in a negative effect on the whole reaction.

#### The reactions of BrO^…^H_2_O + HO_2_ and H_2_O^…^BrO + HO_2_

**Figure 3:**
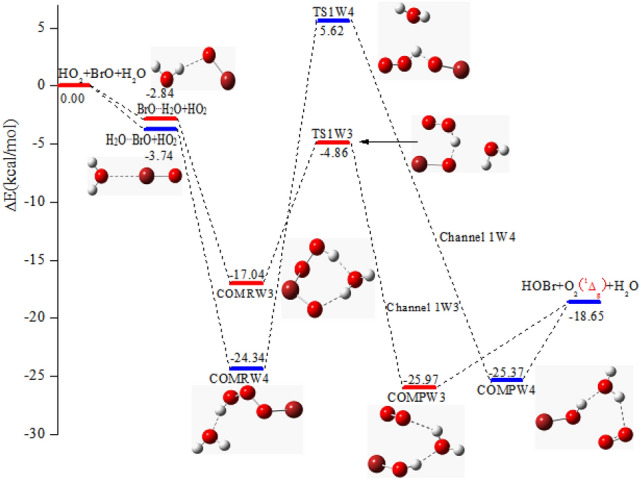
The potential energy surface for the HO_2_ and BrO reaction in the existence of water taking place via BrO^…^H_2_O + HO_2_ and H_2_O^…^BrO + HO_2_ pathway are calculated at the CCSD(T)/aug-cc-pVTZ//B3LYP/6-311 +  + G(d,p) level.

Expect for the above described reaction channels with water, the other two channels were located to generate HOBr + O_2_ (^1^Δ_g_) from the reactions of BrO^…^H_2_O + HO_2_ (Channel 1W3) and H_2_O^…^BrO + HO_2_ (Channel 1W4), which are displayed in Fig. 3. The energy of the halogen bonded complex H_2_O^…^BrO is stable than the hydrogen bond complex BrO^…^H_2_O by 0.90 kcal/mol. Two distinct reaction channels starting from the complexes BrO^…^H_2_O and H_2_O^…^BrO were located.

The complex BrO^…^H_2_O with HO_2_ reaction starts with the generation of the COMRW3 complex possess a lower barrier (12.18 kcal/mol). With respect to BrO^…^H_2_O + HO_2_, the binding energy of complex COMRW3, which have two hydrogen bond structure, is 17.04 kcal/mol below the reactants. Stemming from COMRW3, the O atom in the moiety of BrO in BrO^…^H_2_O extracts the H atom of HO_2_ through TS1W3 (− 4.86 kcal/mol) to generate post-reactive complexe COMPW3 (− 25.97 kcal/mol). In addition, the channel beginning with the generation of COMRW4 proceeds via TS1W4 surmounting a higher barrier (29.96 kcal/mol), which is 17.78 kcal/mol higher than the Channel 1W3. Thus, the H-extraction of Channel 1W4 is much more difficult than that of Channel 1W3. Although Channel 1W3 is the most feasible channel among the BrO + HO_2_ + H_2_O reaction. the barrier of COMRW3 → TS1W3 → COMPW3 in Channel 1W3 is 8.50 kcal/mol higher than the analogous channel without water, which manifested that the introduction of water molecule inhibited the reaction through raising the barrier. In order to validly identificate the influence of water, it is necessary to further research the kinetics of BrO + HO_2_ reaction with and without water molecule.

### Kinetics computations

The above-mentioned mechanism manifested that the existence of a water molecule takes a negative catalytic influence on the BrO + HO_2_ reaction. Water restrains the generation of HOBr + O_2_ (^1^Δ_g_) and also raises the barrier when the reaction occurs via generationn of a transition state. In this work, we execute rate coefficient computations to research the influence of water molecule on the BrO + HO_2_ reaction. At different altitudes, the rate coefficients and the effective rate coefficients for the representative channel of the BrO + HO_2_ reaction both with and without water are summaried in Tables 3 and 4, respectively. Table 3 listed the computed data of the rate coefficient for the channel of generation of HOBr + O_2_ (^1^Δ_g_) and HBr + O_3_ for the BrO + HO_2_ reaction by employing the KisThelP program. The computed rate coefficients for the Channel 1 and Channel 2 in the temperature region of 216.69–298.15 K are 7.16 × 10^–11^–1.44 × 10^–11^ cm^3^ molecule^−1^ s^−1^ and 2.05 × 10^–23^–2.62 × 10^–21^ cm^3^ molecule^−1^ s^−1^, respectively. For the BrO + HO_2_ reaction, the datas of 7.16 × 10^–11^–1.44 × 10^–11^ cm^3^ molecule^−1^ s^−1^ in the researched temperature region are agreement with the previous experimental results^4,10–13^. Larichev et al., Li et al., Bedjanian et al. and Ward and Rowley^4,10,11,13^ measured the rate coefficients at 300 K are 3.29 × 10^–11^, 1.86 × 10^–11^, 2.97 × 10^–11^ and 2.66 × 10^–11^ cm^3^ molecule^−1^ s^−1^, and Cronkhite et al.^12^ measured the rate coefficient at 296 K is 2.01 × 10^–11^ cm^3^ molecule^−1^ s^−1^. Our computed results indicated that the rate coefficients of generating of HOBr + O_2_ (^1^Δ_g_) is 12–9 orders of magnitude faster than that of generation of HBr + O_3_, manifesting that the channel of generating of HOBr + O_2_ (^1^Δ_g_) occupied the BrO + HO_2_ reaction under researched conditions.

**Table 3:** Rate coefficient (in cm^3^ molecule^−1^ s^−1^) for the BrO + HO_2_ reaction without and with one water molecule at different heights (h).

*h* (km)^a^	T (K)^a^	*k*_COMR1_ Channel 1	*k*_COMR2_ Channel 2	*k*_COMRW1_ Channel 1W1	*k*_COMRW2_ Channel 1W2	*k*_COMRW3_ Channel 1W3	*k*_COMRW4_ Channel 1W4
0	298.15	1.44 × 10^–11^	2.62 × 10^–21^	1.29 × 10^–17^	1.99 × 10^–21^	3.83 × 10^–16^	1.98 × 10^–21^
0	288.19	1.66 × 10^–11^	1.67 × 10^–21^	1.12 × 10^–17^	1.33 × 10^–21^	4.16 × 10^–16^	1.11 × 10^–21^
2	275.21	2.03 × 10^–11^	8.90 × 10^–22^	9.12 × 10^–18^	7.61 × 10^–22^	4.72 × 10^–16^	4.89 × 10^–22^
4	262.23	2.55 × 10^–11^	4.42 × 10^–22^	7.30 × 10^–18^	4.11 × 10^–22^	5.38 × 10^–16^	2.00 × 10^–22^
6	249.25	3.28 × 10^–11^	2.06 × 10^–23^	5.73 × 10^–18^	2.09 × 10^–22^	6.26 × 10^–16^	7.47 × 10^–23^
8	236.27	2.36 × 10^–11^	8.84 × 10^–23^	4.40 × 10^–18^	9.87 × 10^–23^	7.45 × 10^–16^	2.51 × 10^–23^
10	223.29	3.60 × 10^–11^	3.46 × 10^–23^	3.27 × 10^–18^	4.28 × 10^–23^	9.00 × 10^–16^	7.44 × 10^–24^
12	216.69	7.16 × 10^–11^	2.05 × 10^–23^	2.79 × 10^–18^	2.30 × 10^–23^	1.01 × 10^–15^	3.80 × 10^–24^

^a^The values are taken from Ref.^48^.

**Table 4:** Effective rate coefficients for the BrO + HO_2_ + H_2_O reaction at 216.69–298.15 K (cm^3^ molecule^−1^ s^−1^).

h (km)^a^	T (K)^a^	[H_2_O]^b^	$k_{{\text{COMR}{1}}}^{^{\prime}}$	$k_{{\text{COMR}{2}}}^{^{\prime}}$	$k_{{\text{COMR}{3}}}^{^{\prime}}$	$k_{{\text{COMR}{4}}}^{^{\prime}}$
0	298.15	7.79 × 10^17^	8.52 × 10^–21^	7.55 × 10^–27^	8.14 × 10^–21^	3.90 × 10^–26^
0	288.19	4.34 × 10^17^	6.12 × 10^–21^	3.15 × 10^–27^	5.99 × 10^–21^	1.51 × 10^–26^
2	275.21	1.89 × 10^17^	3.79 × 10^–21^	9.22 × 10^–28^	3.74 × 10^–21^	3.96 × 10^–27^
4	262.23	7.43 × 10^16^	2.21 × 10^–21^	2.33 × 10^–28^	2.17 × 10^–21^	8.92 × 10^–28^
6	249.25	2.64 × 10^16^	1.21 × 10^–21^	5.13 × 10^–29^	1.19 × 10^–21^	1.72 × 10^–28^
8	236.27	8.15 × 10^15^	6.10 × 10^–22^	9.25 × 10^–30^	5.99 × 10^–22^	2.70 × 10^–29^
10	223.29	2.15 × 10^15^	2.78 × 10^–22^	1.34 × 10^–30^	2.71 × 10^–22^	3.36 × 10^–30^
12	216.69	1.01 × 10^15^	1.77 × 10^–22^	3.90 × 10^–31^	1.74 × 10^–22^	1.04 × 10^–30^

$k_{{\text{COMR}{2}}}^{^{\prime}}$, $k_{{\text{COMR}{1}}}^{^{\prime}}$, $k_{{\text{COMR}{3}}}^{^{\prime}}$ and $k_{{\text{COMR}{4}}}^{^{\prime}}$ are the effective rate coefficients of Channel 1W1, Channel 1W2, Channel 1W3 and Channel 1W4, respectively.

^a^The values are taken from Ref.^48^.

^b^Water concentrations are taken from Ref.^49^.

With the introduction of water, the rate coefficients for the Channel 1W1, Channel 1W2 and Channel 1W4 reveal positive temperature dependence, and the rate coefficients for the Channel 1W3 displays negative temperature dependence. The rate coefficients for the Channel 1W2 and Channel 1W4 are lower than that of Channel 1W1 and Channel 1W3. Moreover, Table 3 indicates that the rate coefficients for Channel 1W1 and Channel 1W3 are much smaller than that for the generation of HOBr + O_2_ in the absence of water at 216.69–298.15 K.

Taking the concentration of the binary complexes HO_2_^…^H_2_O, H_2_O^…^HO_2_, BrO^…^H_2_O and H_2_O^…^BrO into account, it is nessary to compare the effective rate coefficients of the BrO + HO_2_ reaction in the exitence of water with that of in the absence water to fully acquaintancing the influence of water on the BrO + HO_2_ reaction. The rate coefficients for the BrO + HO_2_ reaction in the absence of water could be written as

$$\nu_{{\text{COMR1(or 2)}}} = k_{{\text{COMR1(or 2)}}} \left[ {\text{BrO}} \right]\left[ {\text{HO}_{\text{2}} } \right]$$

whereas the rate coefficients for the generation of HOBr + O_2_ of the BrO + HO_2_ reaction in the existence of water can be written as

$$\nu_{{{\text{COMR1W1}}}} = k_{{\text{COMR}{\text{W1}}}} \left[ {\text{H}_{\text{2}} \text{O} \cdots \text{HO}_{\text{2}} } \right]\left[ {\text{BrO}} \right]{ = }k_{{\text{COMR}{\text{W1}}}}^{^{\prime}} \left[ {\text{HO}_{\text{2}} } \right]\left[ {\text{BrO}} \right]$$

$$\nu_{{{\text{COMR1W1}}}} = k_{{\text{COMR}{\text{W2}}}} \left[ {\text{HO}_{\text{2}} \cdots \text{H}_{\text{2}} \text{O}} \right]\left[ {\text{BrO}} \right]{ = }k_{{\text{COMR}{\text{W2}}}}^{^{\prime}} \left[ {\text{HO}_{\text{2}} } \right]\left[ {\text{BrO}} \right]$$

$$\nu_{{{\text{COMR1W1}}}} = k_{{\text{COMR}{\text{W3}}}} \left[ {\text{BrO} \cdots \text{H}_{2} \text{O}} \right]\left[ {\text{HO}_{2} } \right]{ = }k_{{\text{COMR}{\text{W3}}}}^{^{\prime}} \left[ {\text{HO}_{2} } \right]\left[ {\text{BrO}} \right]$$

$$\nu_{{{\text{COMR1W1}}}} = k_{{\text{COMR}{\text{W4}}}} \left[ {\text{H}_{2} \text{O} \cdots \text{BrO}} \right]\left[ {\text{HO}_{2} } \right]{ = }k_{{\text{COMR}{\text{W4}}}}^{^{\prime}} \left[ {\text{HO}_{2} } \right]\left[ {\text{BrO}} \right]$$

In above equations, $k_{{\text{COMR}{\text{W1}}}}^{^{\prime}} { = }k_{{\text{COMR}{\text{W1}}}} K_{{\text{eq1}}} \left[ {\text{H}_{2} \text{O}} \right]$, $k_{{\text{COMR}{\text{W2}}}}^{^{\prime}} { = }k_{{\text{COMR}{\text{W2}}}} K_{{\text{eq2}}} \left[ {\text{H}_{2} \text{O}} \right]$, $k_{{\text{COMR}{\text{W3}}}}^{^{\prime}} { = }k_{{\text{COMR}{\text{W3}}}} K_{{\text{eq3}}} \left[ {\text{H}_{2} \text{O}} \right]$ and $k_{{\text{COMR}{\text{W4}}}}^{^{\prime}} { = }k_{{\text{COMR}{\text{W4}}}} K_{{\text{eq4}}} \left[ {\text{H}_{2} \text{O}} \right]$. $K_{{\text{eq1}}}$, $K_{{\text{eq2}}}$,$K_{{\text{eq3}}}$ and $K_{{\text{eq4}}}$ are the rate coefficients for the generation of the complexes H_2_O^…^HO_2_, HO_2_^…^H_2_O, BrO^…^H_2_O and H_2_O^…^BrO, respectively. $K_{{\text{eq1}}}$, $K_{{\text{eq2}}}$,$K_{{\text{eq3}}}$ and $K_{{\text{eq4}}}$ are listed in Table S1. The effective rate coefficients of $k_{{\text{COMR}{\text{W1}}}}^{^{\prime}}$, $k_{{\text{COMR}{\text{W2}}}}^{^{\prime}}$, $k_{{\text{COMR}{\text{W3}}}}^{^{\prime}}$ and $k_{{\text{COMR}{\text{W4}}}}^{^{\prime}}$ are decided by the concentration of water to compare the rate coefficient in the absence of water ($k_{{\text{COMR1}}}$ and $k_{{\text{COMR2}}}$), which are given in Table 4. The effective rate coefficients of $k_{{\text{COMR}{\text{W1}}}}^{^{\prime}}$, $k_{{\text{COMR}{\text{W2}}}}^{^{\prime}}$, $k_{{\text{COMR}{\text{W3}}}}^{^{\prime}}$ and $k_{{\text{COMR}{\text{W4}}}}^{^{\prime}}$ at 216.69–298.15 K are 1.77 × 10^–22^–8.52 × 10^–21^ cm^3^ molecule^−1^ s^−1^, 3.90 × 10^–31^–7.55 × 10^–27^ cm^3^ molecule^−1^ s^−1^, 1.74 × 10^–22^–8.14 × 10^–21^ cm^3^ molecule^−1^ s^−1^ and 1.04 × 10^–30^–3.90 × 10^–26^ cm^3^ molecule^−1^ s^−1^, respectively. The computed results reveal that the BrO + HO_2_ reaction in the existence of water are much slower with respect to the feasible channels of the BrO + HO_2_ reaction. In a word, under atmospheric conditions, the above findings manifest that a single water molecule possesses negative influence on the BrO + HO_2_ reaction.

## Conclusion

HOBr is generated through the atmospheric reaction of BrO + HO_2_, which is the temporary storage of BrOx substances. It is great interest to research the influence of water molecule on the mechanism and kinetics of the BrO + HO_2_ reaction. In the present work, the probable catalytic influence of water molecule on the reaction BrO + HO_2_ was reaearched from the perspective of mechanism and kinetics taking advantage of quantum chemical calculation. The rate coefficients at 216.69–298.15 K were obtained by employing the KisThelP program based on the Transition State Theory (TST) with Wigner tunneling correction for the BrO + HO_2_ reaction in the absence and existence water. There exist two distinct channels for the BrO + HO_2_ reaction in the absence water, and the channel of generation of HOBr + O_2_ (^1^Δ_g_) dominant the reaction. With the introduction of water, the influence of a single water was researched through taking into account four distinct types of reactions: HO_2_^…^H_2_O with BrO, H_2_O^…^HO_2_ with BrO, BrO^…^H_2_O with HO_2_, H_2_O^…^BrO with HO_2_. Owning to the higher barrier height, the channel taking palce by BrO^…^H_2_O with HO_2_ may be significant with respect to other channels. The effective rate coefficients of Channle 1W2 and Channle 1W4 are much lower than the reacton in te absence of water. These results come to the conclusion that water molecule inhibits the BrO + HO_2_ reaction through increasing the stability of the pre-reactive complex and raising the barrier. In a word, the present work might contribute to a better comprehending of the influence of water on radical- radical reaction in troposphere.

### Supplementary Information

Supplementary Information.

## Data Availability

The data that support the findings of this study are available on request from the corresponding author.
