# Supplementary material for: The influence of a single water molecule on the reaction of BrO + HO2
Source: Sci Rep. 2023 Aug 10;13:13014. doi: 10.1038/s41598-023-28783-x (PMC10415307; doi:10.1038/s41598-023-28783-x)
Supplement: Supplementary file 1 — Supplementary Information. [file 41598_2023_28783_MOESM1_ESM.docx]

**The influence of a single water molecule on the reaction of BrO + HO_2_**

Peng Zhang^1^, Lu Ma^1^, Meilian Zhao^2^, Yuxi Sun^1^, Wanping Chen^1^, Yunju Zhang,[[1]](#footnote-0)*

*^1^Key Laboratory of Photoinduced Functional Materials, Key Laboratory of Inorganic Materials Preparation and Synthesis, Mianyang Normal University, Mianyang 621000, PR China*

*^2^College of Medical Technology, Chengdu University of Traditional Chinese Medicine Liutai Avenue, Wenjiang District ChengDu PR China*

^1^** Corresponding author. Email address: zhangyj010@nenu.edu.cn Tel.: +86 816 2200064; Fax: +86 816 2200819*

**Table S1.** Calculated equilibrium coefficients for the generation of the HO_2_∙∙∙H_2_O, H_2_O∙∙∙HO_2_, BrO∙∙∙H_2_O and H_2_O∙∙∙BrO (cm^3^ molecule^-1^)

|  | T (K) | *K*_eq1_΄  (H_2_O∙∙∙HO_2_) | *K*_eq2_΄  (HO_2_∙∙∙H_2_O) | *K*_eq3_΄  (BrO∙∙∙H_2_O) | *K*_eq4_΄  (H_2_O∙∙∙BrO) |
| --- | --- | --- | --- | --- | --- |
|  | 298.15 | 8.48×10^-22^ | 4.87×10^-24^ | 2.73×10^-23^ | 2.53×10^-23^ |
|  | 288.19 | 1.26×10^-21^ | 5.46×10^-24^ | 3.32×10^-23^ | 3.14×10^-23^ |
|  | 275.21 | 2.20×10^-21^ | 6.41×10^-24^ | 4.19×10^-23^ | 4.28×10^-23^ |
|  | 262.23 | 4.07×10^-21^ | 7.64×10^-24^ | 5.42×10^-23^ | 6.00×10^-23^ |
|  | 249.25 | 8.03×10^-21^ | 9.29×10^-24^ | 7.20×10^-23^ | 8.72×10^-23^ |
|  | 236.27 | 1.70×10^-20^ | 1.15×10^-23^ | 9.87×10^-23^ | 1.32×10^-22^ |
|  | 223.29 | 3.95×10^-20^ | 1.46×10^-23^ | 1.40×10^-22^ | 2.10×10^-22^ |
|  | 216.69 | 6.29×10^-20^ | 1.68×10^-23^ | 1.71×10^-22^ | 2.71×10^-22^ |

**Table S2.** Computed equilibrium rate coefficients for the generation of the COMRW1, COMRW2, COMRW3 and COMRW4, respectively (cm^3^molecule^-1^).

| T (K) | [H_2_O]^a^ | *K*_eq_(COMRW1) | *K*_eq_(COMRW2) | *K*_eq_(COMRW3) | *K*_eq_(COMRW4) |
| --- | --- | --- | --- | --- | --- |
| 298.15 | 7.79×10^17^ | 2.19×10^-22^ | 3.66×10^-14^ | 6.16×10^-21^ | 2.10×10^-12^ |
| 288.19 | 4.34×10^17^ | 4.03×10^-22^ | 1.33×10^-13^ | 1.41×10^-20^ | 6.99×10^-12^ |
| 275.21 | 1.89×10^17^ | 9.51×10^-22^ | 8.21×10^-13^ | 4.54×10^-20^ | 3.82×10^-11^ |
| 262.23 | 7.43×10^16^ | 2.44×10^-21^ | 6.07×10^-12^ | 1.64×10^-19^ | 2.46×10^-10^ |
| 249.25 | 2.64×10^16^ | 6.92×10^-21^ | 5.52×10^-11^ | 6.78×10^-19^ | 1.93×10^-9^ |
| 236.27 | 8.15×10^15^ | 2.20×10^-20^ | 6.41×10^-10^ | 3.28×10^-18^ | 1.90×10^-8^ |
| 223.29 | 2.15×10^15^ | 8.00×10^-20^ | 9.88×10^-9^ | 1.90×10^-17^ | 2.43×10^-7^ |
| 216.69 | 1.01×10^15^ | 1.64×10^-19^ | 4.51×10^-8^ | 5.04×10^-17^ | 1.00×10^-6^ |

*K*_eq_(COMRW1), *K*_eq_(COMRW2), *K*_eq_(COMRW3) and *K*_eq_(COMRW4) are the computed equilibrium rate coefficients for the generation of binary complexes COMRW1, COMRW2, COMRW3 and COMRW4, respectively. ^a^Water concentrations are taken from Ref 1.[1]

The expression equation for calculating the rate constants of the reactions. The concentration of the particular catalyst is considered and the catalytic effect of the catalyst on the reaction is measured by the effective rate constant. The specific calculation formula is as follows:

In fact, for the BrO + HO_2_ reaction without catalyst, it can occur according to the two-step mechanism proposed in equation 1 and 2:

If *k*_1_ and *k*_-1_ are the forward and reverse rate constants for the first step, respectively, and *k*_2_ corresponds to the second step, the rate constant for this process is shown by eq.3 according to the steady-state conditions.

$$k=\frac{k_{1}k_{2}}{k_{-1}+k_{2}}$$

Due to the very loose transition state, the entropy change in *k*_-1_ is much larger than in the formation of products. Therefore, *k*_-1_ is considerably larger than *k*_2_,^[2-5]^ and a pseudo equilibrium assumption can be used in the formation of the reactant complex. Hence, eq.3 can be represented as

$$k=\frac{k_{1}}{k_{-1}+k_{2}}k_{2}=k_{eq}k_{2}$$

where *K*_eq_ and *k*_2_ are the equilibrium constant of the first step and the rate constant of the second step in the reactions, and they can be displayed by eq. 5 and 6, respectively.

$$k_{eq}=\frac{\sigma Q_{R_{1}\cdots R_{2}}k_{1}k_{2}}{Q_{R_{1}}Q_{R2}}\exp\left(\frac{E_{BrO+HO_{2}}-E_{BrO\cdots HO_{2}}}{RT}\right)$$

$$k_{2}=\frac{\kappa\sigma}{\beta h}\frac{Q_{TS}}{Q_{RC}}\exp\left(-\frac{E_{TS}-E_{BrO\cdots HO_{2}}}{RT}\right)$$

The total rate constant *k* can be obtained as

$$k=k_{2}\frac{\sigma Q_{R_{1}\cdots R_{2}}}{Q_{R_{1}}Q_{R2}}\exp\left(\frac{E_{BrO+HO_{2}}-E_{BrO\cdots HO_{2}}}{RT}\right)$$

where $\sigma$is the symmetry factor; *β* equals the reciprocal of Boltzmann's constant; *h* is Planck's constant; $\kappa$is the tunneling factor; $Q_{ER}$and $Q_{TS}$are partition functions for the reactant complexes and transition state, respectively.

The detailed calculation process of rate coefficients can be referred to the website of <http://sobereva.com/246.>

First, the following input files are used for optimization and vibration analysis of reactant 1, and obtained HO_2_.out

#p b3lyp/6-311++(d,p) opt freq test

Title Card Required

0 2

O -1.19967700 0.64059100 0.01915500

O -0.94535800 -0.66253700 -0.00034900

H 0.04488300 -0.71185600 -0.01631200

Thermodynamic calculation of reactant 2 is performed with the following input file to obtain BrO.out

#p b3lyp/6-311++(d,p) opt freq test

Title Card Required

0 2

Br 0.00000000 0.00000000 0.32677100

O 0.00000000 0.00000000 -1.42962100

Use the following input files to search the transition state structure and conduct vibration analysis to obtain TS1.out

#p b3lyp/6-311++g(d,p) opt=(calcfc,ts,noeigentest) freq test

TS1

0 1

O -3.05555559 1.45502643 0.00000000

H -1.80555559 1.45502643 0.00000000

O -3.49618064 2.69931320 0.00000000

Br -0.67039931 3.71210057 -0.41359653

O -0.57036548 1.91488684 -0.41760095

The calculation of equilibrium rate constant is based on the website of <http://sobereva.com/310>

Reference:
[1] Lowe, P. R. An approximating polynomial for the computation of saturation vapor pressure. J. Appl. Meteorol. 1977, 16, 100-103.

[2] Singleton, D. L.; Cvetanovic, R. J. Temperature Dependence of CH_3_O_2_•+HO_2_•→CH_3_O_2_H + O_2_ reaction in the gas phase. Computational Evidence for the Formation of a Hydrogen-Bonded Diradical Complex. J. Phys. Chem. A 2006, 110, 6073−6082.

[3] Galano, A.; Alvarez-Idaboy, J. R.; Ruiz-Santoyo, M. E.; Vivier-Bunge, A. Glycolaldehyde + OH Gas Phase Reaction: A Quantum Chemistry + CVT/SCT Approach. J. Phys. Chem. A 2005, 109, 169−180.

[4] Alvarez-Idaboy, J. R.; Mora-Diez, N.; Vivier-Bunge, A. A Quantum Chemical and Classical Transition State Theory Explanation of Negative Activation Energies in OH Addition To Substituted Ethenes. J. Am. Chem. Soc. 2000, 122, 3715−3720.

[5] Sun, H.; Law, C. K. Kinetics of Hydrogen Abstraction Reactions of Butene Isomes by OH Radical. J. Phys. Chem. A 2010, 114, 12088−12098.

**Figure S1** Optimized geometries of reactants, pre-reactive complexes, transition states, and post-reactive complexes for the HO_2_ + BrO and HO_2_ + BrO + HO_2_ reaction at the B3LYP/6-311++G(d,p) level of theory.

TS1

O 1.97875400 0.23243800 -0.17964800

H 1.34017900 0.99088700 0.21511700

O 1.39860000 -0.88817700 0.16602300

Br -0.79985800 -0.19406400 -0.02625200

O -0.04550000 1.38090800 0.10158600

TS2

O 1.52979700 1.07685000 -0.15912800

H 0.27262400 0.82362500 -0.23485900

O 1.97531600 -0.08498300 0.32043300

Br -1.13447200 -0.00461000 0.01729600

O 1.42412400 -1.07465400 -0.20761700

TS1W1

O 0.96830300 1.44005500 0.34227700

H 1.13805500 0.37934100 0.09943400

O -0.11254500 1.77177200 -0.27200000

Br -1.23051300 -0.35541100 -0.03103100

O 0.45082400 -0.80078400 0.28374700

O 3.12954900 -0.62774400 -0.24928700

H 3.82872600 -0.76503200 0.39734600

H 2.61212000 -1.44132100 -0.24859100

TS1W2

O -0.26578000 1.63857200 -0.14071900

H 0.19582100 0.44113300 0.00717800

O -1.56400400 1.41027100 -0.30454100

Br 0.12658600 -1.21308900 -0.04341400

O -2.01419400 0.52733200 0.45888400

O 2.50863000 1.35718000 0.19071500

H 2.99644700 1.98096400 -0.35614400

H 3.06000400 0.56915700 0.23374500

TS1W3

O 0.96906400 1.43942400 0.34253000

H 1.13814600 0.37839900 0.09985700

O -0.11134800 1.77187500 -0.27204800

Br -1.23061200 -0.35505900 -0.03115000

O 0.45042900 -0.80112400 0.28443200

O 3.12862400 -0.62821200 -0.24966400

H 3.82741400 -0.76492400 0.39751400

H 2.61169000 -1.44211900 -0.24913100

TS1W4

O -1.55176700 -0.97132100 -0.43049500

H -1.17824700 -0.01185600 -0.15128300

O -2.61955800 -1.24496400 0.17092100

Br 1.57482100 -0.01759300 -0.07673200

O -2.00018400 1.77222200 -0.20377800

H -2.91708500 1.93969900 0.03726600

H -1.46492200 2.16685500 0.49363700

O -0.02330300 0.00919500 0.75159900

COMR1

O 2.54301900 -0.54692500 -0.00726400

H 0.76855900 0.56180000 0.02646400

O 3.55874900 0.10023100 0.00578900

O -0.03814000 1.10251400 -0.00529400

Br -1.40793100 -0.16595300 0.00079100

COMR2

O -2.25724700 -0.64876900 -0.22961700

H -1.96773900 -1.39434000 0.32338000

O -1.60956900 0.45905900 0.43635000

Br 1.07158900 -0.11922000 0.01684200

O -0.57541700 0.88558800 -0.32083900

COMRW1

O 1.35899700 -1.47230000 -0.36087600

H 1.93874200 -0.67463900 -0.21785800

O 0.35219700 -1.28555800 0.56790400

Br -1.03722900 -0.01182800 -0.06445800

O -0.36842200 1.55574700 0.08730500

O 2.39358000 0.98837800 -0.06347700

H 2.93131300 1.33257000 0.65614700

H 1.54214500 1.46590100 -0.02910700

COMRW2

O 1.82258700 -1.04161400 -0.55818700

H 2.09523800 -0.11051900 -0.37024500

O 0.93000200 -1.28017900 0.54066600

Br -1.27051900 0.37363400 -0.04121500

O -0.32774900 -1.37508400 0.08442300

O 2.32882900 1.58888800 0.04306500

H 2.86652300 1.86730300 0.79174600

H 1.47703300 2.02993600 0.14127800

COMRW3

O 1.36106200 -1.47887700 -0.35677200

H 1.94607500 -0.69036400 -0.19468500

O 0.34367000 -1.29272100 0.56125500

Br -1.03340900 -0.01056900 -0.07389800

O -0.37656300 1.55924300 0.10516800

O 2.38362700 0.97566500 0.08248800

H 2.99190000 1.49181300 -0.45452700

H 1.53696500 1.46199600 0.09851000

COMRW4

O -1.94053100 1.20136400 -0.67608800

H -2.62037100 0.48741000 -0.63532900

O -1.35821600 1.10893500 0.62153100

O -3.35677900 -1.08185100 -0.08961900

H -3.78023300 -1.76939300 -0.61277000

H -2.59625300 -1.49121800 0.34228100

O -0.69979100 -0.08800300 0.74991900

I 1.27998900 -0.11981800 -0.07434200

COMP1

O 2.53455200 -0.17371200 0.49565600

H 0.72172900 0.79647900 0.12650800

O 3.32516800 -0.21279300 -0.41210500

Br -1.32848500 -0.21414900 -0.01707500

O -0.13781300 1.22384700 -0.02466300

COMP2

O -1.90743200 -0.99318400 0.01252600

H 0.27821400 -0.18449100 0.04726200

O -2.87965300 -0.19255500 -0.02068000

Br 1.68955100 0.03949900 -0.00165600

O -2.63947700 1.03599100 0.00949300

COMPW1

O 1.75311200 1.19720000 0.46068400

H 0.60596200 -1.22667300 0.50981400

O 0.99551200 1.70817900 -0.35237000

Br -1.23756600 -0.03052200 -0.14968600

O -0.06264000 -0.75534100 1.06609000

O 2.07845300 -1.55210300 -0.37977000

H 2.44190200 -0.65464100 -0.33094900

H 2.15146000 -1.83391300 -1.29690600

COMPW2

O -2.51296000 0.15828400 -0.54706100

H 0.72103400 0.97917300 0.11850500

O -2.12944600 -0.99826600 -0.17112300

Br 1.51109100 -0.23872200 -0.08504300

O -1.31309600 -1.06237400 0.77461700

O -0.51298300 2.23188500 0.31591800

H -1.33985100 1.75123400 0.15899300

H -0.52150900 2.98864500 -0.27979400

COMPW3

O 1.71150600 1.24041700 0.45484300

H 0.65781000 -1.21196000 0.43266500

O 0.91685200 1.75499500 -0.31974500

Br -1.24979200 -0.06096900 -0.14225700

O -0.02225700 -0.79346000 1.01613400

O 2.11720300 -1.45154100 -0.50205300

H 2.49014000 -0.56922600 -0.35202800

H 2.80833500 -2.08817700 -0.29507300

COMPW4

O 1.36106200 -1.47887700 -0.35677200

H 1.94607500 -0.69036400 -0.19468500

O 0.34367000 -1.29272100 0.56125500

Br -1.03340900 -0.01056900 -0.07389800

O -0.37656300 1.55924300 0.10516800

O 2.38362700 0.97566500 0.08248800

H 2.99190000 1.49181300 -0.45452700

H 1.53696500 1.46199600 0.09851000

HO_2_…H_2_O

O -0.77651200 0.62339500 -0.00058500

O -1.53360900 -0.46384100 0.00133400

O 2.13610800 -0.22274800 -0.00138400

H 1.20917000 0.05258300 0.00284700

H 2.63995500 0.59597500 0.00753900

H -2.45701800 -0.14300900 -0.00530900

H_2_O…HO_2_

O -0.94514600 -0.66258400 -0.00033700

H 0.04512400 -0.71195600 -0.01754700

O -1.19920500 0.64065900 0.01931700

O 1.66418400 0.00765600 -0.08083400

H 2.37251000 -0.11551200 0.55887300

H 1.42370000 0.94162100 -0.04649500

BrO…H_2_O

Br 0.93689900 -0.15581100 0.00000000

O -0.36245400 1.01879100 -0.00000200

O -2.99023900 -0.38664100 -0.00000100

H -3.75170700 0.20019500 -0.00004500

H -2.21822300 0.19598200 0.00008800

H_2_O…BrO

O -2.51082400 0.00001200 -0.03099000

H -3.05299400 0.76923000 0.17089800

H -3.05307700 -0.76913600 0.17094000

O 2.04211200 0.00003700 0.03300700

Br 0.28159300 -0.00001400 -0.01022800

BrO

Br 0.00000000 0.00000000 0.32677100

O 0.00000000 0.00000000 -1.42962100

HO_2_

O 0.05526900 0.71882600 0.00000000

O 0.05526900 -0.60930200 0.00000000

H -0.88430000 -0.87619300 0.00000000

H_2_O

O 0.00000000 0.00000000 0.11704100

H 0.00000000 0.76348700 -0.46816500

H 0.00000000 -0.76348700 -0.46816500

HOBr

H -0.92047900 1.70476100 0.00000000

Br 0.02140600 -0.38769300 0.00000000

O 0.02140600 1.48306100 0.00000000

HBr

H 0.00000000 0.00000000 -1.38700300

Br 0.00000000 0.00000000 0.03962900

O_3_

O 1.07935100 -0.21449500 0.00000000

O 0.00000000 0.42832200 0.00000000

O -1.07935100 -0.21382700 0.00000000

O_2_ (^1^Δ_g_)

O 0.00000000 0.00000000 0.60271600

O 0.00000000 0.00000000 -0.60271600

**Examples of the GAUSSIAN inputs of IRC searches for the important species (TS1 and TS1W1).**

#p b3lyp/6-311++g(d,p) irc=(calcfc,,maxpoint=30) iop(5/13=1) test nosymm

TS1-IRC

0 1

O 1.97875400 0.23243800 -0.17964800

H 1.34017900 0.99088700 0.21511700

O 1.39860000 -0.88817700 0.16602300

Br -0.79985800 -0.19406400 -0.02625200

O -0.04550000 1.38090800 0.10158600

#p b3lyp/6-311++g(d,p) irc=(calcfc,maxpoint=50) iop(5/13=1) test nosymm

TS1W2-IRC

0 1

O -0.99994100 1.39230000 -0.30140000

H -1.14056300 0.34290600 -0.00409700

O 0.11278500 1.76471800 0.23181800

Br 1.24904300 -0.34081500 0.01801100

O -0.43669500 -0.83966800 -0.14350100

O -3.17797400 -0.60293000 0.03985200

H -3.78067200 -0.65146000 0.78826400

H -2.78069300 -1.47828600 -0.02868900

1. [↑](#footnote-ref-0)
